# Cost drivers associated with autologous stem-cell transplant (ASCT) in patients with relapsed/refractory diffuse large B-cell lymphoma in a Japanese real-world setting: A structural equation model (SEM) analysis 2012–2022

**DOI:** 10.1371/journal.pone.0317439

**Published:** 2025-02-06

**Authors:** Saaya Tsutsué, Shinichi Makita, Hiroya Asou, Shingo Wada, Wen Shi Lee, Dilinuer Ainiwaer, Koki Idehara, Sona-Sanae Aoyagi, Seok-Won Kim, Todd Taylor

**Affiliations:** 1 Kite Value and Access, Gilead Sciences Japan, Tokyo, Japan; 2 National Cancer Center Hospital, Tokyo, Japan; 3 Kite Clinical Development, Gilead Sciences, Tokyo, Japan; 4 RWES, IQVIA Solutions Japan, K.K., Tokyo, Japan; Istanbul University-Cerrahpaşa, Cerrahpaşa Faculty of Medicine, TÜRKIYE

## Abstract

Diffuse large B-cell lymphoma (DLBCL) is the most prevalent non-Hodgkin lymphoma, with increasing incidence, in Japan. It is associated with substantial economic burden and relatively poor survival outcomes for relapsed/ refractory (r/r) DLBCL patients. Despite its association with economic burden and the relatively limited number of eligible patients in Japan as reported in previous real-world studies, Japanese clinical guidelines recommend stem-cell transplantation (SCT) for transplant-eligible r/r DLBCL patients. This is the first study to elucidate the total healthcare cost, associated cost drivers and healthcare resource use of SCT among patients with r/r DLBCL in a nationwide setting. The study design included a follow-up period of up to 24 months with subsequent lines of therapies using retrospective nationwide claims data from the Medical Data Vision Co., Ltd. Health Insurance Association from April 2012 to August 2022. Included patients had a confirmed diagnosis of DLBCL, received allogeneic SCT (allo-SCT) or autologous SCT (ASCT) after the first DLBCL diagnosis, and received high-dose chemotherapy during the 6-month look-back period. The results confirmed that no patients had allo-SCT, hence only ASCT was included in the analysis. Structural equation modeling was used to identify potential total healthcare cost drivers by evaluating direct, indirect, and total effects and provide a benchmark reference for future innovative therapies. A total of 108 patients (3.8%) among all DLBCL patients who received SCT met the eligibility criteria and were considered ASCT patients; majority of which were males (n = 63, 58.33%), with a mean [median] (SD) age of 52.04 [55] (9.88) years. A total of 15 patients (13.89%) received subsequent therapies. The most frequent subsequent therapy was GDP-based with or without rituximab (n = 8, 7.41%). The mean [median] (SD) number of follow-up hospitalizations on or after SCT-related hospitalizations was 1.66 [1] (1.36), with a mean [median] (SD) length of hospital stay being 36.88 [34] (12.95) days. The total mean [median] (SD) healthcare cost after adjustment incurred per patient per year during follow-up was $79,052.44 [$42,722.82] ($121,503.65). Number of hospitalizations and Charlson Comorbidity Index scores (+5) were the key drivers of total healthcare costs in patients with r/r DLBCL. Index years 2020–2022 and heart disease as a complication were other statistically significant factors that had positive effects as increase on total healthcare costs.

## Introduction

Non-Hodgkin lymphoma (NHL), a heterogeneous group of lymphoid malignancies, is the most prevalent hematological malignancy globally [1]. In Japan, 34,873 new cases of NHL were reported in 2019, and the number of patients steadily increased during 2016 to 2019 [2]. In Japan, NHL accounts for 39.6% of all hematological malignancies, and the incidence has steadily increased from 1993 to 2008 [3,4]. DLBCL accounts for approximately 35.8% of NHL cases in Japan [5,6].

In terms of epidemiological features, approximately 30%–40% of patients with DLBCL have disease progression during or at the end of the initial chemotherapy (refractory) or remission (relapse) after the initial response [7,8]. According to Japanese clinical guidelines, treatment with high-dose salvage chemotherapy and autologous stem-cell transplantation (ASCT) is recommended for patients with transplant-eligible, chemotherapy-sensitive r/r DLBCL. However, nearly 50% of the transplant-eligible patients have a response with high-dose salvage chemotherapy before undergoing ASCT, with an overall curative rate in the range of 25%–35% [9].

In a retrospective cohort study conducted in the US, total all-cause costs were 17.6% higher in the SCT group than the non-SCT group [10]. In the US, patients received R-CHOP as primary treatment. However, around 28% of the patients received SCT as 2L approach, which further increased the healthcare cost. The cost of treatment for year 1 was 210,488 United States dollars (USD) and for year 2 was 267,770 USD [11]. In a population-based observational study conducted in the United Kingdom, the expected total medical costs during the SCT-related hospitalization period and all follow-up periods were £22,122 for those treated with curative intent, and the treatment cost increased with the change in line of treatment [12]. In Japan, the estimated mean [median] (SD) costs of the 2L and 3L treatments including any SCT-related costs was $73,296.40 [$58,223.11] ($58,409.79) and $75,238.35 [$60,477.31] ($59,583.66), respectively [5]. The mean [median] (SD) total cost for 2L during the follow-up and at the index time in Japan was reported as $77,173.5 [$62,848.0] ($59,643.2) and $33,210.1 [$28,056.8] ($25,559.1), respectively, with inpatient visits being the highest contributor [13].

Nevertheless, understanding the cost drivers responsible for the high burden on patients with r/r DLBCL is limited, especially in the Japanese clinical setting where patients are likely to receive follow-up in different hospital settings for SCT as can be seen in the SCT cost data [3,5]. Furthermore, the advent of innovative therapies has led to a major paradigm shift in the management of patients with r/r DLBCL. Improvement in patient profiles in terms of complete response, median progression-free survival, and overall survival has been reported for patients treated with chimeric antigen receptor T-cell (CAR T) versus alternate therapies [14,15]. In 2019, axicabtagene ciloleucel (axi-cel), tisagenlecleucel (tisa-cel), and lisocabtagene maraleucel (liso-cel) were launched and reimbursed in Japan for patients including r/r DLBCL after two or more lines of systemic therapy; however, only axi-cel and liso-cel were launched and reimbursed for r/r DLBCL following one prior line of therapy in 2022 [16]. Still those therapies are not widely used in Japan in the post CAR T cell therapy era, and future work is necessary to conduct similar cost driver analysis [5] inclusive of CAR T cell therapies. Data will be accumulated to consider optimal care for patients in post CAR T cell therapy era.

Japan has nationwide universal health insurance coverage that can provide a nationwide snapshot of the current treatment landscape through health insurance database analysis. Furthermore, to unveil potential cost drivers and factors driving direct health care costs in patients with r/r DLBCL receiving SCT in Japan, it is imperative to capture continuous follow-up data across multiple medical providers. As opposed to our study which uses health insurance claims data, a previous study using hospital-based data was unable to analyze full patient care histories for patients who transferred to different medical institutions because follow-up across institutions is not possible [5]. From the standpoint of the Japanese health care system, this data could serve as a benchmark for optimizing patient care in Japan to consider innovative therapies that may be further introduced in a Japanese public co-payment reimbursement setting. Therefore, in this study, the primary objective is to estimate the potential cost drivers associated with SCT in r/r DLBCL patients. This study used structural equation modeling (SEM) to elucidate cost drivers in patients with r/r DLBCL during SCT-related hospitalization and follow-up period.

## Methods

### Study design and data source

This retrospective observational study of patients with DLBCL receiving SCT was conducted using claims from the nationwide Medical Data Vision Co., Ltd. (MDV; Tokyo, Japan) Health Insurance Association (HIA) database from April 2012 to August 2022 inclusive. The MDV HIA database is a payer-based health care claims database that provides information on patient demographics and clinical aspects, including diagnoses coded according to the International Classification of Diseases Tenth Revision (ICD-10) codes and Japanese standard disease codes, prescriptions, and medical treatment. The database includes inpatient, outpatient, and pharmacy claims from 149 health insurance societies in Japan, with approximately 7.84 million unique identifiers, and covers about 3.0% of the Japanese population (as of August 2022) [17].

**Fig 1:**
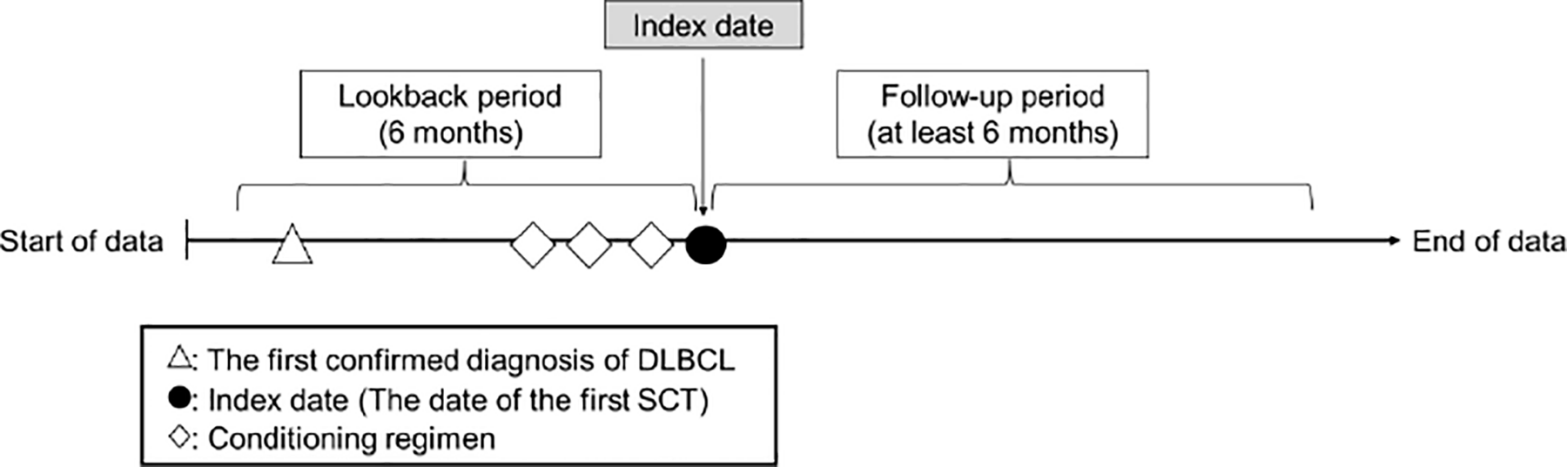
Study design.

The MDV HIA database includes patient demographics such as age, diagnoses coded according to ICD-10 codes and the Japanese standard disease codes, prescriptions, procedures, and dates of hospital admission and discharge. The database is unlinked and anonymized and has been used for epidemiological and health economics and outcomes research [5]. The requirement of informed consent for our analyses was waived due to the anonymous nature of the database. The index date was defined as the date of the first SCT. An overview of the analysis design is presented in Fig 1.

### Study population

Patients eligible for the study had a confirmed diagnosis of DLBCL based on the ICD-10 codes (C83.3, C83.8, C85.1, C85.2) at least once during October 2012 to February 2022, received allo-SCT or ASCT (medical procedure codes: 150225910, 150297810, 150266310, and 150266410) after the confirmed diagnosis of DLBCL, and received high-dose chemotherapy (conditioning regimen) during the 6-month look-back period [3,5] in order to secure sufficient number of patients to conduct SEM analysis.

Patients receiving CAR T therapy before the index date (Anatomical Therapeutic Chemical classification code: L01X5), not having a 6-month look-back period, or not having at least a 6-month follow-up period were excluded from the cohort in this study.

### Ethical considerations

In accordance with the Ethical Guidelines for Epidemiological Research issued by the Japanese Ministry of Health, Labour and Welfare, ethics approval and informed consent were not applicable for this study. Institutional Review Board (IRB)/Independent Ethics Committee (IEC) review was not required for this secondary analysis of existing data since patient identifiers in all data sources had been removed by the database vendor (MDV). Anonymized structured data were used that did not involve any interventions.

### Study variables and outcomes

Study variables and outcomes were defined based on each study objective, and the primary objective was to determine the cost drivers among patients with r/r DLBCL receiving SCT. Other outcomes of interest included treatment patterns of subsequent therapies after the first SCT, health care resource utilization (HCRU), and the associated costs. Different categories of therapies have been elaborated in S1 Table. Of note, Yescarta, a Gilead CAR T regimen, was not included in the analysis due to data availability in this dataset.

HCRU-related outcomes measured included the number of hospitalizations during the follow-up period starting from the first SCT-related hospitalization (inclusive); the percentage of patients with intensive care unit (ICU) admission, positron emission tomography (PET) scans, magnetic resonance imaging (MRI) scans, computerized tomography (CT) scans, emergency room (ER) admission, and radiation therapy; and the length of hospital stay (LOS) during the first SCT-related hospitalization. The corresponding health care costs were estimated using the latest unit prices for the medical services (as of April 2022) and the drugs (as of April 2023) for which had been listed at the time. Otherwise, the unit prices were adjusted to those as of April 2022 by the revision rates of the biannual medical service fee and drug price revisions, in line with Japanese HTA C2H guidelines [18]. The admission date for SCT-related hospitalization was defined as the first date of hospitalization wherein the patient received SCT based on the start of consecutive records of basic hospitalization fees. The discharge date for the first SCT-related hospitalization was defined as the last consecutive record of basic hospitalization fees related to the hospitalization.

Sociodemographic and clinical variables included gender, age, age group, the geographical location of the medical facility where the patient received SCT, the comorbidities of the patients—including modified Charlson Comorbidity Index (CCI) score categories, index year groups, the length of the follow-up period, prior/concurrent non-lymphoma neoplasms, and complications regarding heart, kidney, and liver diseases acquired after SCT [5].

### Statistical analysis

For general considerations, descriptive statistics were summarized for the baseline characteristics and other outcomes of interest. Frequencies and percentages were reported as categorical variables, while means with standard deviations (SDs), medians, minimum and maximum values, and 25 and 75 percentile values were reported as continuous variables, when applicable. Health care costs were reported on a per-patient, per-year basis. The following formula was used for the calculation of health care costs:


$$\left[Healthcarecost\left(perpatientperyear\right)\right]=\frac{\left[Relevantcostsincurredduringtheperiod\right]}{\left[Totallengthoftheperiodindays\right]}\times 365\left(days\right)$$

Cost drivers within multiple parameters (captured from the MDV HIA database) in the study were determined using SEM by evaluating direct effects, indirect effects, and total effects simultaneously in the model [5]. The following formulas were used:

Direct effects:


$$\left[totalhealthcarecost\left(THCC\right)\right]\sim w\text{* }patientcharacteristics+x\text{* }\left[comorbidities\right]+y\text{* }\left[subsequenttherapy\right]+v\text{* }\left[complications\right]+z\text{* }\left[HCRU\right]$$

Indirect effects:


$$patientcharacteristics\left(indirect\right):=a\text{* }y+b\text{* }z+c\text{* }v$$

$$comorbidities\left(indirect\right):=d\text{* }y+e\text{* }v+f\text{* }z$$

Total effects:


$$total,patientcharacteristics:=w+patientcharacteristics\left(indirect\right)$$

$$total,comorbidities:=x+comorbidities\left(indirect\right)$$

total,subsequenttherapy:=y

total,complications:=v

total,HCRU:=z

The relationship between the total health care cost related to the first SCT-related hospitalization and cost factors (i.e., patient baseline characteristics and comorbidities) was calculated using the following formula:


$$\left[Totalhealthcarecost\right]=\sim w\text{* }\left[Patientbaselinecharacteristics\right]+x\text{* }\left[Comorbidities\right]$$

where “=~” indicates the measurement model.

Model evaluation assesses model performance or fit based on the fit indices for the test of a single path coefficient, such as a two-sided test for statistical significance (p-values), coefficients (B), standard coefficients (β), β with 95% CIs, and overall model fits. Goodness-of-fit for each model was measured by the standardized root mean square residual (SRMR), wherein a value less than 0.08 was considered a well-fitted model.

#### Model conceptualization and finalization.

The first conceptual model (Model 0) was constructed, and parameters (e.g., patient characteristics, comorbidities [i.e., CCI score and complications]) were included based on the study by Tsutsué et al., 2022 [5]. Then, Model 0 was modified to find a goodness-of-fit model based on a standardized root mean squared residual (RMSA < 0.08). This was determined by categorizing each variable according to the objectives of the study (Models 0, 1, and 2 in S2–S4 Tables). Model 1 was adapted from model 0 by incorporating changes in the variable, chemotherapy regimens post-SCT wherein any subsequent therapy post-SCT was considered. Although RMSA was 0.059, concerns regarding combining chemotherapy with different costs were present. Model 2 was then developed by modifying model 1 where chemotherapy regimens post-SCT was stratified into CAR T cell therapy and any other chemotherapy. The RMSA was 0.056 but concerns regarding grouping regimens other than CAR T were persistent. In particular, some regimens (such as Bendamustine) were often performed without hospitalization, hence that might affect total healthcare costs. This led to the development of the final model. In Model 3, patients were segregated into 3 categories based on chemotherapy regimen post-SCT with CAR T, with outpatient regimen and without any regimen. There was an overlap seen among patients with different reference for each regimen. Finally, goodness-of-fit was achieved in Model 3 based on SRMR.

Regarding the categorization of variables in the final model, chemotherapy regimens post-SCT were categorized as outpatient regimens, CAR T cell therapy, and other regimens, while in the conceptual model (Model 0) chemotherapy regimens comprised Rituximab (R) +/− dexamethasone, etoposide, ifosfamide, and carboplatin (DeVIC)-based, R-CHASE-based, GDP-based with or without R, R-bendamustine-based, R-EPOCH, DA-EPOCH, DA-EPOCH-R, R +/− ESHAP-based, R- ifosfamide (ICE)-based, R-DHAP-based, Pola-BR, Pola-R-CHP, and CAR T cell therapy. The analysis was performed using R software (version 4.3.1) with the lavaan package (version 0.6.16).

## Results

### Patient characteristics, treatment patterns, health care resource utilization, and costs

#### Baseline characteristics of patients.

**Fig 2:**
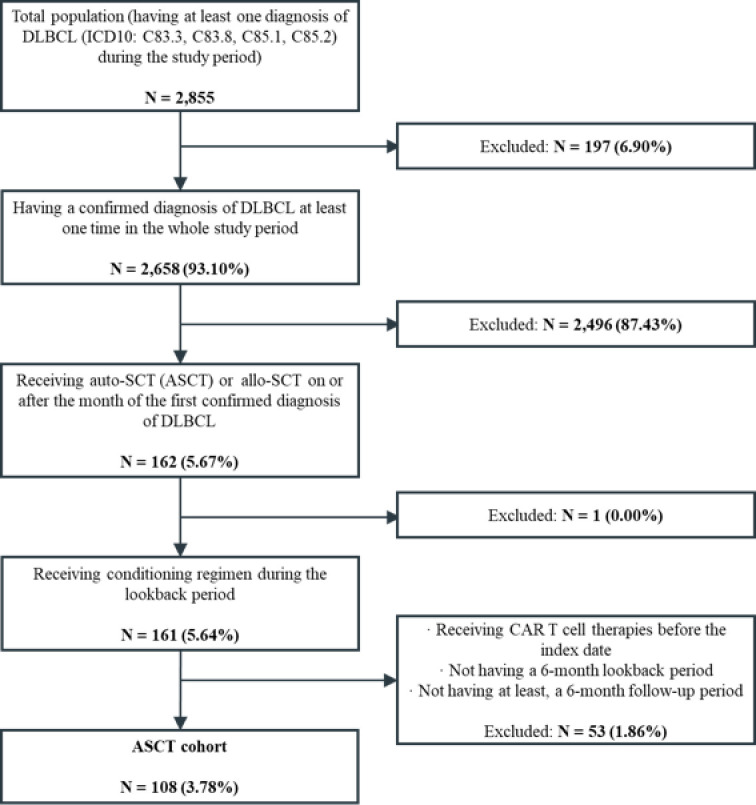
Patient attrition.

A total of 2,855 patients diagnosed with DLBCL were identified from the database of which 108 (3.8%) who underwent SCT were included in the analysis. All patients underwent ASCT as the initial SCT and no patient had allo-SCT identified in result. The patient attrition is presented in Fig 2.

The mean [median] (SD) age of the patients was 52.04 [55] (9.88) years with the majority of the patients categorized to the age group of 18-65 years (n = 105, 97.2%). There were more male patients (n = 63, 58.33%) in the study population (Table 1). It is notable that the group with index years between 2012 and 2019 inclusive, had a higher patient burden (n = 74, 68.52%) versus the group with index years between 2020 and 2022 inclusive (n = 34, 31.48%). The mean [median] (SD) length of follow-up among patients with r/r DLBCL was 926.63 [686.5] (695.09) days. Follow-up was defined from the index date until death or the last patient record. Approximately 40% of the patients received ASCT at medical facilities (n = 42, 38.89%) in the Kanto region of Japan (S5 Table). With respect to clinical characteristics, nearly half of the patients (n = 49, 45.37%) had a modified CCI score of ≥ 5, with liver disease accounting for the most prevalent complication (n = 8, 7.41%). The majority (n = 66, 61.11%) of the patients had prior/concurrent non-lymphoma neoplasms.

**Table 1:** Patient demographics and clinical characteristics.

Patient characteristics	N = 108
**Gender n (%)**	
Male; Female	63 (58.33); 45 (41.67)
Age at index date	
**Mean (SD)**	52.04 (9.88)
Median (Q1, Q3) (Min, Max)	55 (48, 58) (22, 71)
**Age groups n (%)**	
< 18 years	0 (0.00)
18–65 years	105 (97.22)
≥66 years	3 (2.78)
**Index year groups (inclusive) n (%)**	
2012–2019	74 (68.52)
2020–2022	34 (31.48)
**Length of follow-up period (days)**	
Mean (SD)	926.63 (695.09)
Median (Q1, Q3) (Min, Max)	686.5 (397.5, 1275.5) (206, 3098)
**Modified CCI score categories n (%)**	
0–2	14 (12.96)
3	17 (15.74)
4	28 (25.93)
5+	49 (45.37)
**Prior/concurrent non-lymphoma neoplasms n (%)**	
Yes; No	66 (61.11); 42 (38.89)
**Complications n (%)**	
Heart disease	4 (3.70)
Kidney disease	2 (1.85)
Liver disease	8 (7.41)

Abbreviations: CCI, Charlson comorbidity index; Max, maximum; Min, minimum; Q1, first quartile; Q3, third quartile; SD, standard deviation.

A total of 15 patients (13.89%) were initiated on subsequent therapy post-SCT. Notedly, the patient count was not mutually exclusive, i.e., patients could be started on more than 1 therapy at any specific time. The most frequently prescribed subsequent therapy was GDP-based with or without R (n = 8, 7.41%), followed by R + /-DeVIC-based (n = 4, 3.70%) (Table 2). Similar prescription patterns were observed for R-CHASE-based, CAR T cell therapy, R-EPOCH, DA-EPOCH, DA-EPOCH-R, R + /-ESHAP-based, and pola-BR (n = 2, 1.85% for each). The least-prescribed regimens were R-bendamustine-based therapy (n = 1, 0.93%) and R-ICE-based therapy (n = 1, 0.93%). No regimen was observed for R-DHAP-based therapy or Pola-R-CHP in subsequent therapies after ASCT.

**Table 2:** Subsequent therapies received by patients with DLBCL post-ASCT.

Subsequent therapies	n (%)
R+/-DeVIC-based	4 (3.70)
R-CHASE-based	2 (1.85)
GDP-based with or without R	8 (7.41)
R-bendamustine-based	1 (0.93)
R-EPOCH, DA-EPOCH, DA-EPOCH-R	2 (1.85)
R+/-ESHAP-based	2 (1.85)
R-ICE-based	1 (0.93)
R-DHAP-based	0 (0.00)
Pola-BR	2 (1.85)
Pola-R-CHP	0 (0.00)
CAR T cell therapy	2 (1.85)

Abbreviations: CAR T cell, chimeric antigen receptor T cell; CHASE, cyclophosphamide, cytarabine, etoposide, dexamethasone; DA, dose-adjusted; DeVIC, dexamethasone, etoposide, ifosfamide, carboplatin; DHAP, dexamethasone, cytarabine, cisplatin; ESHAP, etoposide, cytarabine, cisplatin, methylprednisolone; EPOCH, etoposide, prednisolone, vincristine, cyclophosphamide, doxorubicin; GDP, gemcitabine, dexamethasone, cisplatin/carboplatin; ICE, ifosfamide, carboplatin, etoposide; Pola-BR, polatuzumab vedotin, bendamustine, and rituximab; Pola-R-CHP, polatuzumab vedotin, rituximab, cyclophosphamide, doxorubicin, and prednisone; R, rituximab.

#### Health care resource utilization.

The mean [median] (SD) number of follow-up hospitalizations during or after the ASCT-related hospitalization was 1.66 [1] (1.36), with no ICU or ER admissions after the first hospitalization (Table 3). Nearly half of the patients (n = 55, 50.93%) had a CT scan at least once, while 3 (2.78%) underwent a PET scan, 12 (11.11%) had an MRI scan, and only 1 was subjected to radiation therapy during ASCT-related hospitalization. The mean (SD) LOS of the first SCT-related hospitalization was 36.88 (12.95).

**Table 3:** HCRU findings.

HCRU	N = 108
**Number of follow-up hospitalizations (for any reason) during or after SCT-related hospitalization**	
Mean (SD)	1.66 (1.36)
Median (Q1, Q3)	1 (1, 2)
**Any ICU admission during SCT-related hospitalization, n (%)**	
Yes, No	0 (0.00), 108 (100.00)
**Any PET scans during SCT-related hospitalization, n (%)**	
Yes, No	3 (2.78), 105 (97.22)
**Any MRI scans during SCT-related hospitalization, n (%)**	
Yes, No	12 (11.11), 96 (88.89)
**Any CT scans during SCT-related hospitalization, n (%)**	
Yes, No	55 (50.93), 53 (49.07)
**Any emergency room visits during SCT-related hospitalization, n (%)**	
Yes, No	0 (0.00), 108 (100.00)
**Any radiation therapies during SCT-related hospitalization, n (%)**	
Yes, No	1 (0.93), 107 (99.07)
**LOS of SCT-related hospitalizations**	
Mean (SD),	36.88 (12.95)
Median (Q1, Q3) (Min, Max)	34 (28.5, 42) (12, 84)

Abbreviations: CT, computed tomography; HCRU, health care resource utilization; ICU, intensive care unit; LOS, length of hospital stay; MRI, magnetic resonance imaging; PET, positron emission tomography; Q1, first quartile; Q3, third quartile; SCT, stem-cell transplantation; SD, standard deviation.

#### Health care costs.

The total mean [median] (SD) health care cost incurred per patient per year during the follow-up period was $79,052.44 [$42,722.82] ($121,503.65) after adjustment and $78,671.67 [$42,792.39] ($120,747.97) before adjustment (Table 4). Health care costs in Japanese yen (JPY) are presented in Supplementary Information (S6 Table). The unit prices were adjusted to those as of April 2022 by the revision rates of the biannual medical service fee and drug price revisions (which were accounted for inflation as well as global health care budget) (S7 Table), in line with Japanese HTA C2H guidelines with following USD conversion method from previous study [19].

**Table 4:** Total health care costs per patient per year in USD.

N = 108	Cost before adjustment*		Cost after adjustment*	
Mean, SD	$78,671.67	$120,747.97	$79,052.44	$121,503.65
Median	$42,792.39	–	$42,722.82	–
Q1, Q3	$21,395.94	$73,708.08	$21,475.21	$73,722.68
Min, Max	$5,144.37	$707,137.74	$5,166.36	$705,239.70

*Adjustment of medical fees and drug prices to those as of April 2022.

Abbreviations: JPY, Japanese yen; Q1, first quartile; Q3, third quartile; USD, United States dollars.

### Structural equation model

**Fig 3:**
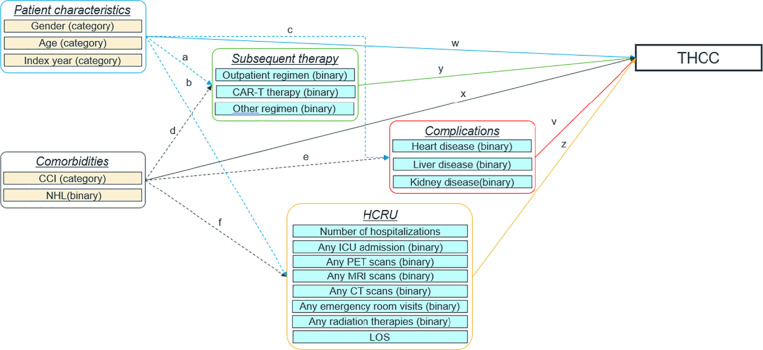
Conceptual framework of Model 4.

The conceptual model (Model 0) was modified in terms of study objectives to yield the final model (Model 4) with an SRMR of 0.077, which is deemed a good fitting model in SEM [19]. The final conceptual framework of SEM pathways representing the relationships between the total health care cost and potential cost factors is shown in Fig 3.

**Fig 4:**
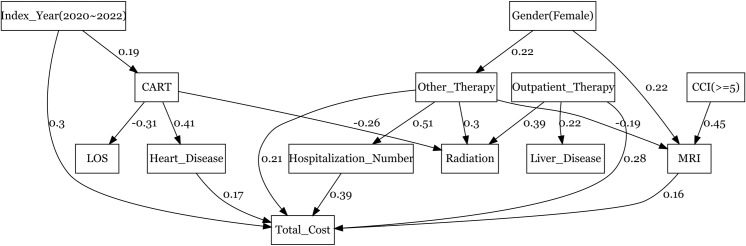
SEM with path analysis for cost drivers representing statistically significant effects of direct and indirect paths.

The number of hospitalizations had the greatest impact on the total health care cost in terms of the total effect (β: 0.389 [95% CI: 0.222, 0.555]). CCI score ≥ 5 (β: 0.355, [95% CI: 0.069, 0.641]) and receiving “other regimens” post-ASCT (β: 0.328, [95% CI: 0.164, 0.493]) were other key drivers of the total health care cost. “Other regimens” included any of the following chemotherapies: Pola-R-CHP, R + /-DeVIC-based, R-CHASE-based, R + /-ESHAP-based, R-ICE-based, R-DHAP-based, and R-EPOCH, DA-EPOCH, and DA-EPOCH-R (excluding outpatient regimens and CAR T cell therapies). Index years 2020–2022 (baseline was 2012–2019) (β: 0.188, [95% CI: 0.009, 0.368]), heart disease as a complication (β: 0.174, [95% CI: 0.013, 0.334]), and outpatient regimens during the subsequent chemotherapy (β: 0.217, [95% CI: 0.055, 0.379]) were also statistically significant factors through mediators on the total health care cost. However, Length of Stay (LOS) did not significantly influence the total health care cost. The model coefficients for the total, direct, and indirect effects are presented in Table 5, while Fig 4 presents the significant factors of the model.

**Table 5:** Direct, indirect, and total effects on health care cost obtained from Model 4 (best-fit model).

Total health care cost drivers	N = 108	Direct effects (USD)			Indirect effects (USD)			Total effects (USD)		
	n (%)	β	95% CI	p	β	95% CI	p	β	95% CI	p
**Patient characteristics**										
Gender (reference: male)										
Female	45 (41.67)	−0.079	−0.234; 0.075	0.313	0.118	−0.006; 0.242	0.061	0.039	−0.143; 0.221	0.676
Age (reference: 18–65 years)										
≥66 years	3 (2.78)	-0.002	−0.151; 0.147	0.976	−0.060	−0.178; 0.057	0.315	−0.062	−0.245; 0.120	0.502
Index year (reference: 2012–2019)										
2020–2022	34 (31.48)	0.299	0.148; 0.450	<0.001***	−0.110	−0.240; 0.019	0.095	0.188	0.009; 0.368	**0.039** *
**Comorbidities**										
CCI score (reference: 0–2)										
3	17 (15.74)	−0.001	−0.207; 0.205	0.995	0.090	−0.074; 0.254	0.283	0.089	−0.158; 0.336	0.479
4	28 (25.93)	0.149	−0.073; 0.371	0.188	0.050	−0.126; 0.227	0.579	0.199	−0.069; 0.468	0.146
5+	49 (45.37)	0.174	-0.078; 0.427	0.175	0.180	−0.020; 0.381	0.078	0.355	0.069; 0.641	**0.015** *
**Prior/concurrent non-lymphoma neoplasms (reference: No)**										
Yes	66 (61.11)	−0.079	−0.238; 0.079	0.326	0.020	−0.108; 0.148	0.759	−0.059	−0.250; 0.132	0.542
**Complications**										
Heart disease (reference: No)	4 (3.70)	0.174	0.013; 0.334	**0.034** *	–	–	–	0.174	0.013; 0.334	**0.034** *
Kidney disease (reference: No)	2 (1.85)	0.031	−0.115; 0.176	0.678	–	–	–	0.031	−0.115; 0.176	0.678
Liver disease (reference: No)	8 (7.41)	-0.056	−0.204; 0.092	0.459	–	–	–	−0.056	−0.204; 0.092	0.459
**Chemotherapy regimen post-SCT**										
Chemotherapy regimen post-SCT†										
Outpatient regimen	9 (8.33)	0.285	0.121; 0.448	<0.001***	−0.067	−0.182; 0.048	0.252	0.217	0.055; 0.379	**0.009** **
CAR T cell therapy	2 (1.85)	0.026	−0.150; 0.202	0.775	0.121	−0.006; 0.248	0.061	0.147	−0.020; 0.313	0.084
Other regimens	8 (7.41)	0.206	0.019; 0.392	**0.031** *	0.122	−0.016; 0.261	0.083	0.328	0.164; 0.493	<0.001***
**HCRU**										
Number of hospitalizations	–	0.389	0.222; 0.555	<0.001***	–	–	–	0.389	0.222; 0.555	<0.001***
Any ICU admission (reference: No)*	0 (0.00)	–	–	–	–	–	–	–	–	–
Any PET scans	3 (2.78)	−0.005	−0.152; 0.143	0.949	–	–	–	−0.005	−0.152; 0.143	0.949
Any MRI scans	12 (11.11)	0.161	0.003; 0.318	**0.046** *	–	–	–	0.161	0.003; 0.318	**0.046** *
Any CT scans	55 (50.93)	−0.053	−0.205; 0.099	0.494	–	–	–	−0.053	−0.205; 0.099	0.494
Any emergency room visits*	0 (0.00)	–	–	–	–	–	–	–	–	–
Any radiation therapies	1 (0.93)	−0.127	−0.302; 0.047	0.153	–	–	–	−0.127	−0.302; 0.047	0.153
LOS**	–	0.133	−0.022; 0.288	0.093	–	–	–	0.133	−0.022; 0.288	0.093

Abbreviations: CAR T, chimeric antigen receptor T cell; CCI, Charlson Comorbidity Index; CI, confidence interval; CT, computed tomography; HCRU, health care resource utilization; ICU, intensive care unit; LOS, length of hospital stay; MRI, magnetic resonance imaging; PET, positron emission tomography; SCT, stem-cell transplantation; SD, standard deviation; USD, United States dollars.

*No variance in the variable, therefore, no effects observed.

**Log link has been applied for total healthcare costs drivers in SEM.

‡Hu and Bentler, 1999: SRMR of < 0.08 represents a well-fitted model.

Factors displaying a statistically significant direct effect included index years 2020–2022 (baseline was 2012–2019) (β: 0.299, [95% CI: 0.148, 0.450], p-value: 0.000), heart disease as a complication (β: 0.174, [95% CI: 0.013, 0.334], p-value: 0.034), and outpatient regimens (β: 0.285, [95% CI: 0.121, 0.448], p-value: 0.001). Other cost drivers imposing a direct effect were the number of hospitalizations (β: 0.389, [95% CI: 0.222, 0.555], p-value: 0.000), other regimens as subsequent therapy (β: 0.206, [95% CI: 0.019, 0.392], p-value: 0.031), and MRI scans during the first SCT hospitalization (β: 0.161, [95% CI: 0.003, 0.318], p-value: 0.000).

Other regimens as subsequent therapy (β: 0.122, [95% CI: -0.016, 0.261], p-value: 0.083), CAR T cell therapy as subsequent therapy (β: 0.121, [95% CI: -0.006, 0.248], p-value: 0.061), and CCI score ≥ 5 (β: 0.180, [95%CI: -0.020, 0.381], p-value: 0.078) were major cost drivers indirectly influencing the total healthcare cost. However, statistical significance was not observed for any cost variable in indirect effect determination.

## Discussion

To the best of our knowledge, this study is the first nationwide study to examine the total cost, associated cost driver and health care resource use associated with ASCT among patients with r/r DLBCL in Japan with a follow-up period of 24 months. The previous study conducted by Tsutsué et al., 2022 using the MDV database focused on treatment patterns, patient survival rates, healthcare costs, and economic burden in patients with DLBCL using Japanese Diagnosis, Procedure Combination (DPC) claims database [3,13]. Moreover, the Tsutsué et al., 2022 study identified the cost drivers associated with r/r DLBCL in Japan targeting combination of salvage chemotherapy regimens and SCT cohorts [5]. However, we used HIA claims database in order to fully capture appropriate total healthcare costs and focus on ASCT in this study. This specific population group is often related to higher cost burden as reported in a previous study [11]; hence, this study holds significance in providing clinical insights via elucidation of potential cost drivers and cost reducers in ASCT which will be the benchmark reference material to assess innovative therapies from forward looking perspectives [17,18].

The mean [median] (SD) total healthcare cost during the follow-up in a retrospective analysis amounted to $79,052.44 [$42,722.82] ($121,503.65). The total costs incurred for 2L and 3L chemotherapies were $77,173.5 and $79,118.0, respectively versus the total cost for ASCT in this study ($78,671.7) [13]. Although the cost of DLBCL treatment in Japan is not directly comparable with other regions owing to the difference in healthcare systems. However, few global studies have been included to highlight the cost per se. A prior study suggests a slightly higher treatment cost for DLBCL in the US, where the mean health care cost for 1L, 2L, and 3L was $111,314, $88,472, and $103,365, respectively [20] The mean cost per-patient-per-month for DLBCL in the US during follow-up was $11,890 which further decreased in the subsequent year attributable to low inpatient admissions and decrease in outpatient and chemotherapy services [21]. A separate study established higher mean cost of index procedure for CAR T cell therapy in comparison to SCT ($371,136 CAR T, $96,515 auto-SCT, $169,269 allo-SCT) [22]. In a real-world setting, the time-unadjusted absolute cost for DLBCL for different treatment lines were €59,868 (43,331) for 1L, €35,870 (37,387) for 2L, and €28,832 (40,540) for 3L with hospitalizations (71% of total costs) and drug acquisition costs (18% of total costs) being the primary cost drivers [23]. The average cost per inpatient as observed in a tertiary healthcare setting in Germany was €44,750 for 2L, €32,589 for 3L and €88,668 for> 3L of treatment. The mean treatment cost per-patient for auto-SCT was €55,468 [24]. However, in the US, the outpatient cost were the main drivers of the total healthcare cost in DLBCL patients at $88,202 per-patient-per-year [25].

Another potential cost driver that had a positive relationship with total health care costs in this study was a CCI score ≥ 5. In the study conducted by Tsutsué et al., 2022, it was reported that the CCI score was a statistically significant cost driver with a direct effect on health care costs. The study results were consistent with the previous studies conducted in Japan [3,5,13]. However, this finding did not translate into statistical significance in the total effect [5]. In terms of SEM, since the parameters in this study were different from the previous SEM analysis conducted by Tsutsué et al., 2022, differences in the result of these studies could be expected. Since CCI scores represent the health of patients, it is also expected that higher CCI scores would be associated with higher treatment costs. The study findings confirmed hospitalization frequency, higher CCI scores (5+), and that receiving specific chemotherapy regimens after ASCT are the primary cost drivers in patients with DLBCL receiving ASCT. For treatments after ASCT, GDP-based treatments with or without R were the most frequently used treatment regimens. With respect to complications, heart disease also had a direct positive effect on total health care costs in the study, a finding that was in line with a previous study [5].

HCRU is another key parameter that influences health care cost through a direct effect. This study used SEM to determine the structural relationship between patient characteristics, HCRU, and total healthcare cost. SEM analysis revealed the number of hospitalizations to be the greatest cost driver, causing a direct effect through a surge in the total health care cost. Among HCRU-related drivers, the cost of any MRI scans, number of hospitalizations, and LOS positively affected total health care costs. Furthermore, whether the regimens are conducted in outpatient or inpatient settings also alters the cost outcomes. In a previous nationwide claims database study conducted in Japan, the mean number of hospitalizations during 2L and 3L chemotherapies was 5.1 and 5.0, respectively, with the mean LOS for 2L and 3L chemotherapies being 140.0 and 144.9 days, respectively with different cohort analyzed comprised of SCT and salvage chemotherapy regimens [13]. Hospitalization-related resource utilization for 2L and 3L chemotherapies in the study was higher than the resource utilization for ASCT in our study. However, the number of hospitalizations was not as impactful in the study conducted by Tsutsué et al., 2022 [5]. Regarding HCRU and healthcare costs, the patients were hospitalized 1.66 times per-year (including SCT-related hospitalization), with an average LOS of 36.88 days. This might be because the previous study included different cohorts for analysis with different patterns of subsequent therapy regimen types due to launch and reimbursement sequence timing in the Japanese universal public co-payment system.

Another parameter in this study that was a cost driver of the total health care cost without going through mediators was the index year. Index year ≥ 2020 was treated as a binary variable in our study (i.e., “from 2020 to 2022” with reference “from 2012 to 2019”). However, in the reference study by Tsutsué et al., 2020, the index year was not a cost driver of the total healthcare costs (i.e., it showed an opposite result to this study) and was included as a continuous variable (from 2008 to 2019) [5] and also previous Tsutsué et al., 2020 did not included CAR T cell therapies. Thus, the variables were different between the two studies, and the index year periods were different, i.e., after 2020 was included in this study. The findings of this study are suggestive of a probable treatment paradigm shift around 2020; however further investigation is warranted as potential future work to further elucidate this point.

Additionally, in this study, 13.89% patients received subsequent therapy. Regarding chemotherapy regimens post-ASCT in our study, CAR T cell therapy had no effect on total health care costs, although both outpatient and other regimens had a positive effect on total health care costs without going through mediators (i.e., indirect path). However, since only 2 patients received CAR T cell therapy, this result may not be a representation of the current scenario in Japan and therefore future analysis is required when CAR T data are accumulated in this HIA dataset. Furthermore, the proportion of elderly population in Japan (≥65 years old) represents around 28.1% of the total population as of the 2018 census [26]. This population group is often not considered as eligible auto-SCT candidate [27]. This will further reduce the clinical applicability of auto-SCT in Japan. The recommended age per treatment consensus for eligibility for auto-SCT is 65 years and early 70s for allogeneic stem-cell transplant (allo-SCT) for those who are neither eligible for auto-SCT transplant nor failed SCT [3]. In the current study, 97.2% of 108 patients were eligible to receive ASCT since they were in the age group pf 18-85 years. However, in the previous Tsutsué et al., 2020 study, the mean age of the patients diagnosed with DLBCL was 69.9 years from the 1L to the 5L. This highlights the major unmet medical needs in Japan where patients attain the age of transplant non-eligibility at the time of diagnosis. Such patients should receive innovative therapies in order to obtain potential cure. In the ZUMA-7 study, the chemoimmunotherapy along with ASCT was given as standard of care while axi-cel, a CAR-T was used as 2L of therapy. The median age of the patients were 59 years. The axi-cel group showed significant improvements in the event-free and overall survival in comparison to the standard of care [3,5,13,28,29]. The findings from the study analysis may serve as a benchmark when considering new innovative therapies for patients with r/r DLBCL in a form of cost-utility model parameters [30] and understanding snapshot of current care burden of ASCT.

Despite providing substantial information on cost drivers for patients with DLBCL in Japan, the analysis was limited in certain aspects. A general limitation associated with the database is selection bias. Additionally, only 2 patients were on CAR T cell therapy, increasing the possibility of sample size bias within the study. Further study is required in the future on the association of accumulation of CAR T data to be analyzed for its potential effect in the post CAR T era. Due to the attribute of HIA compared to DPC, the database provides information primarily on relatively younger population (i.e., working-age company employees and their family members). In addition, health insurance members who transferred to other health insurance associations (due to changing jobs or retirement) were not followed up after the transfer. Furthermore, the HIA database does not contain detailed clinical information to identify DLBCL patients by disease severity or disease progression and other details other than claiming for the health insurance bureau.

## Conclusion

To the best of our knowledge, this is the first study to examine the potential cost driver and economic burden among patients with r/r DLBCL receiving ASCT in Japan. Identifying the cost drivers of ASCT-related costs in patients with r/r DLBCL may provide insights for the perspective of optimized patient care.

## Supporting information

S1 Table Categories of therapy. (DOCX)

S2 Table Direct, indirect, and total effects on health care cost obtained from Model 0. (DOCX)

S3 Table Direct, indirect, and total effects on health care cost obtained from Model 1. (DOCX)

S4 Table Direct, indirect, and total effects on healthcare cost obtained from Model 2. (DOCX)

S5 Table Geographical distribution of hospital facilities conducting ASCT. (DOCX)

S6 Table Total health care costs per patient per year in JPY and USD. (DOCX)

S7 Table Medical fee price index for recent medical service fee revisions. (DOCX)
