# Supplementary material for: Cost drivers associated with autologous stem-cell transplant (ASCT) in patients with relapsed/refractory diffuse large B-cell lymphoma in a Japanese real-world setting: A structural equation model (SEM) analysis 2012–2022
Source: PLoS One. 2025 Feb 6;20(2):e0317439. doi: 10.1371/journal.pone.0317439 (PMC11801729; doi:10.1371/journal.pone.0317439)
Supplement: S1 Table — (DOCX) [file pone.0317439.s001.docx]

**S1 Table: Categories of therapy**

| Subsequent therapy regimen | Rituximab (R)+/- dexamethasone etoposide, ifosfamide, carboplatin (DeVIC)-based, R- cyclophosphamide, cytarabine, etoposide, dexamethasone (CHASE)-based, gemcitabine, dexamethasone, cisplatin/carboplatin (GDP-based) with or without R, R-bendamustine-based, rituximab, etoposide phosphate, prednisone, vincristine, cyclophosphamide, hydroxydaunorubicin (doxorubicin) (R- [EPOCH]), dose-adjusted EPOCH (DA-EPOCH), dose-adjusted R-EPOCH (DA-EPOCH-R), etoposide, cytarabine, cisplatin, methylprednisolone with or without R (R+/- [ESHAP]-based, ifosfamide, carboplatin, etoposide with R (R-ICE-based), dexamethasone, cytarabine, cisplatin with or without R (R- [DHAP]-based, polatuzumab vedotin, bendamustine and rituximab (Pola-BR), polatuzumab vedotin rituximab, cyclophosphamide, doxorubicin, and prednisone (Pola-R-CHP), and chimeric antigen receptor (CAR)-T cell therapy |
| --- | --- |
| Outpatient Regimen | GDP-based with or without R, R-bendamustine-based, and Pola-BR |
| Other Regimen | R+/-DeVIC-based, R-CHASE-based, R-EPOCH/DA-EPOCH/DA-EPOCH-R, R+/-ESHAP-based, R-ICE-based, R-DHAP-based, and Pola- R-CHP |
