# Supplementary material for: Cost drivers associated with autologous stem-cell transplant (ASCT) in patients with relapsed/refractory diffuse large B-cell lymphoma in a Japanese real-world setting: A structural equation model (SEM) analysis 2012–2022
Source: PLoS One. 2025 Feb 6;20(2):e0317439. doi: 10.1371/journal.pone.0317439 (PMC11801729; doi:10.1371/journal.pone.0317439)
Supplement: S2 Table — (DOCX) [file pone.0317439.s002.docx]

**S2 Table: Direct, indirect, and total effects on health care cost obtained from Model 0**

| **Total health care cost drivers** | **N=108** | **Direct effects (USD)** | | | **Indirect effects (USD)** | | | **Total effects (USD)** | | |
| --- | --- | --- | --- | --- | --- | --- | --- | --- | --- | --- |
|  | **N (%)** | **β** | **95% CI** | **p** | **β** | **95% CI** | **p** | **β** | **95% CI** | **p** |
| **Patient characteristics** | | | | | | | | | | |
| Gender (reference: male) | | | | | | | | | | |
| Female | 45 (41.67) | -0.071 | -0.223; 0.080 | 0.358 | 0.109 | -0.030; 0.248 | 0.123 | 0.038 | -0.144; 0.221 | 0.681 |
| Age (reference: 18–65 years) | | | | | | | | | | |
| ≥66 years | 3 (2.78) | 0.002 | -0.139; 0.143 | 0.974 | -0.064 | -0.192; 0.064 | 0.327 | -0.062 | -0.244; 0.121 | 0.509 |
| Index year (reference: 2012–2019) | | | | | | | | | | |
| 2020–2022 | 34 (31.48) | 0.299 | 0.152; 0.447 | **<0.001***** | -0.114 | -0.257; 0.029 | 0.118 | 0.186 | 0.006; 0.365 | **0.043*** |
| **Comorbidities** | | | | | | | | | | |
| CCI score (reference: 0–2) | | | | | | | | | | |
| 3 | 17 (15.74) | -0.017 | -0.217; 0.183 | 0.867 | 0.105 | -0.078; 0.288 | 0.260 | 0.088 | -0.160; 0.335 | 0.487 |
| 4 | 28 (25.93) | 0.096 | -0.124; 0.316 | 0.391 | 0.100 | -0.101; 0.300 | 0.329 | 0.196 | -0.073; 0.465 | 0.153 |
| 5+ | 49 (45.37) | 0.175 | -0.072; 0.421 | 0.165 | 0.175 | -0.049; 0.398 | 0.125 | 0.349 | 0.063; 0.636 | **0.017*** |
| **Prior/concurrent non-lymphoma neoplasms (reference: No)** | | | | | | | | | | |
| Yes | 66 (61.11) | -0.091 | -0.244; 0.063 | 0.249 | 0.032 | -0.110; 0.174 | 0.658 | -0.058 | -0.250; 0.133 | 0.549 |
| **Complications** | | | | | | | | | | |
| Heart disease (reference: No) | 4 (3.70) | 0.192 | 0.030; 0.354 | **0.020*** | - | - | - | 0.192 | 0.030; 0.354 | **0.020*** |
| Kidney disease (reference: No) | 2 (1.85) | 0.021 | -0.116; 0.159 | 0.761 | - | - | - | 0.021 | -0.116; 0.159 | 0.761 |
| Liver disease (reference: No) | 8 (7.41) | -0.099 | -0.253; 0.055 | 0.207 | - | - | - | -0.099 | -0.253; 0.055 | 0.207 |
| **Chemotherapy regimen post-SCT** | | | | | | | | | | |
| Chemotherapy regimen post-SCT † | | | | | | | | | | |
| R+/-DeVIC-based | 4 (3.70) | 0.079 | -0.134; 0.292 | 0.468 | 0.067 | -0.118; 0.253 | 0.476 | 0.146 | -0.012; 0.305 | 0.071 |
| R-CHASE-based | 2 (1.85) | -0.021 | -0.183; 0.142 | 0.800 | 0.212 | 0.093; 0.331 | **<0.001***** | 0.191 | 0.029; 0.354 | **0.021*** |
| GDP-based without or without R | 8 (7.41) | 0.274 | 0.116; 0.431 | **<0.001**** | -0.061 | -0.181; 0.059 | 0.318 | 0.213 | 0.056; 0.369 | **0.008**** |
| R-Bendamustine-based | 1 (0.93) | -0.220 | -0.431; -0.009 | **0.041*** | 0.177 | -0.004; 0.358 | 0.055 | -0.043 | -0.209; 0.123 | 0.609 |
| R-EPOCH, DA-EPOCH, DA-EPOCH-R | 2 (1.85) | 0.206 | 0.041; 0.372 | **0.014*** | 0.049 | -0.076; 0.175 | 0.442 | 0.256 | 0.097; 0.415 | **0.002**** |
| R+/-ESHAP-based | 2 (1.85) | 0.007 | -0.144; 0.158 | 0.926 | -0.048 | -0.154; 0.059 | 0.382 | -0.040 | -0.203; 0.122 | 0.627 |
| R-ICE-based | 1 (0.93) | 0.103 | -0.035; 0.242 | 0.144 | 0.005 | -0.083; 0.094 | 0.904 | 0.109 | -0.053; 0.270 | 0.188 |
| R-DHAP-based* | 0 (0.00) | - | - | - | - | - | - | - | - | - |
| Pola-BR | 2 (1.85) | 0.142 | -0.002; 0.287 | 0.054 | -0.006 | -0.104; 0.091 | 0.897 | 0.136 | -0.026; 0.298 | 0.100 |
| Pola-R-CHP* | 0 (0.00) | - | - | - | - | - | - | - | - | - |
| CAR T cell therapy | 2 (1.85) | 0.106 | -0.119; 0.330 | 0.356 | 0.030 | -0.167; 0.227 | 0.767 | 0.135 | -0.026; 0.297 | 0.101 |
| **HCRU** | | | | | | | | | | |
| Number of hospitalizations | - | 0.434 | 0.275; 0.593 | **<0.001***** | - | - | - | 0.434 | 0.275; 0.593 | **<0.001***** |
| Any ICU admission (reference: No)* | 0 (0.00) | - | - | - | - | - | - | - | - | - |
| Any PET scans | 3 (2.78) | -0.009 | -0.148; 0.131 | 0.903 | - | - | - | -0.009 | -0.148; 0.131 | 0.903 |
| Any MRI scans | 12 (11.11) | 0.153 | 0.001; 0.305 | **0.049*** | - | - | - | 0.153 | 0.001; 0.305 | **0.049*** |
| Any CT scans | 55 (50.93) | -0.058 | -0.228; 0.111 | 0.499 | - | - | - | -0.058 | -0.228; 0.111 | 0.499 |
| Any emergency room visits* | 0 (0.00) | - | - | - | - | - | - | - | - | - |
| Any radiation therapies | 1 (0.93) | -0.145 | -0.423; 0.133 | 0.307 | - | - | - | -0.145 | -0.423; 0.133 | 0.307 |
| LOS** | - | 0.176 | -0.016; 0.368 | 0.072 | - | - | - | 0.176 | -0.016; 0.368 | 0.072 |

Abbreviations: SD, standard deviation; CCI, Charlson Comorbidity Index; R, rituximab; DA, dose-adjusted; DeVIC, dexamethasone, etoposide, ifosfamide, carboplatin; CHASE, cyclophosphamide, cytarabine, etoposide, dexamethasone; GDP, gemcitabine, dexamethasone, cisplatin/carboplatin; EPOCH, etoposide, prednisolone, vincristine, cyclophosphamide, doxorubicin; ESHAP, etoposide, cytarabine, cisplatin, methylprednisolone; ICE, ifosfamide, carboplatin, etoposide; DHAP, dexamethasone, cytarabine, cisplatin; Pola-BR, polatuzumab vedotin, bendamustine, and rituximab; Pola-R-CHP, polatuzumab vedotin rituximab, cyclophosphamide, doxorubicin, and prednisone; CAR T cell, chimeric antigen receptor T-cell; CI, confidence interval; CT, computed tomography; HCRU, health care resource utilization; ICU, intensive care unit; LOS, length of hospital stay; MRI, magnetic resonance imaging; PET, positron emission tomography; SD, standard deviation; SCT, stem-cell transplantation.

*No variance in the variable, therefore, no effects observed

**Log link has been applied for total heathcare costs drivers in sem.

‡Hu and Bentler,1999: SRMR of <0.08 represents a well-fitted model
