# Supplementary material for: Cost drivers associated with autologous stem-cell transplant (ASCT) in patients with relapsed/refractory diffuse large B-cell lymphoma in a Japanese real-world setting: A structural equation model (SEM) analysis 2012–2022
Source: PLoS One. 2025 Feb 6;20(2):e0317439. doi: 10.1371/journal.pone.0317439 (PMC11801729; doi:10.1371/journal.pone.0317439)
Supplement: S3 Table — (DOCX) [file pone.0317439.s003.docx]

**S3 Table: Direct, indirect, and total effects on health care cost obtained from Model 1**

| **Total health care cost drivers** | **N = 108** | **Direct effects (USD)** | | | **Indirect effects (USD)** | | | | **Total effects (USD)** | | |
| --- | --- | --- | --- | --- | --- | --- | --- | --- | --- | --- | --- |
|  | **N (%)** | **β** | **95% CI** | **p** | **β** | **95% CI** | **p** | | **β** | **95% CI** | **p** |
| **Patient characteristics** | | | | | | | | | | | |
| Gender (reference: male) | | | | | | | | | | | |
| Female | 45 (41.67) | -0.079 | -0.229; 0.071 | 0.303 | 0.116 | -0.009; 0.241 | 0.068 | | 0.038 | -0.145; 0.220 | 0.687 |
| Age (reference: 18–65 years) | | | | | | | | | | | |
| ≥66 years | 3 (2.78) | -0.004 | -0.151; 0.142 | 0.956 | -0.056 | -0.178; 0.065 | 0.362 | | -0.060 | -0.244; 0.123 | 0.517 |
| Index year (reference: 2012–2019) | | | | | | | | | | | |
| 2020–2022 | 34 (31.48) | 0.309 | 0.165; 0.453 | **<0.001***** | -0.127 | -0.254; 0.001 | 0.052 | | 0.182 | 0.002; 0.363 | **0.047*** |
| **Comorbidities** | | | | | | | | | | | |
| CCI score (reference: 0–2) | | | | | | | | | | | |
| 3 | 17 (15.74) | -0.007 | -0.205; 0.191 | 0.946 | 0.093 | -0.070; 0.257 | 0.263 | | 0.086 | -0.162; 0.334 | 0.495 |
| 4 | 28 (25.93) | 0.140 | -0.077; 0.357 | 0.205 | 0.052 | -0.128; 0.233 | 0.570 | | 0.193 | -0.077; 0.462 | 0.161 |
| 5+ | 49 (45.37) | 0.170 | -0.075; 0.416 | 0.174 | 0.173 | -0.031; 0.377 | 0.096 | | 0.344 | 0.056; 0.631 | **0.019*** |
| **Prior/concurrent non-lymphoma neoplasms (reference: No)** | | | | | | | | | | | |
| Yes | 66 (61.11) | -0.071 | -0.228; 0.085 | 0.371 | 0.014 | -0.118; 0.146 | 0.835 | | -0.057 | -0.249; 0.134 | 0.557 |
| **Complications** | | | | | | | | | | | |
| Heart disease (reference: No) | 4 (3.70) | 0.180 | 0.036; 0.324 | **0.014*** | - | - | - | | 0.180 | 0.036; 0.324 | **0.014*** |
| Kidney disease (reference: No) | 2 (1.85) | 0.036 | -0.106; 0.179 | 0.618 | - | - | - | | 0.036 | -0.106; 0.179 | 0.618 |
| Liver disease (reference: No) | 8 (7.41) | -0.054 | -0.198; 0.089 | 0.459 | - | - | - | | -0.054 | -0.198; 0.089 | 0.459 |
| **Chemotherapy regimen post-SCT** | | | | | | | | | | | |
| Chemotherapy regimen post-SCT † | | | | | | | | | | | |
| Any subsequent chemotherapy (reference: No) | 15 (13.89) | 0.329 | 0.167; 0.491 | **<0.001***** | 0.114 | 0.000; 0.229 | **0.050*** | | 0.444 | 0.293; 0.594 | **<0.001***** |
| **HCRU** | | | | | | | | | | | |
| Number of hospitalizations | - | 0.378 | 0.226; 0.529 | **<0.001***** | - | - | | - | 0.378 | 0.226; 0.529 | **<0.001***** |
| Any ICU admission (reference: No)* | 0 (0.00) | - | - | - | - | - | | - | - | - | - |
| Any PET scans | 3 (2.78) | -0.001 | -0.145; 0.143 | 0.993 | - | - | | - | -0.001 | -0.145; 0.143 | 0.993 |
| Any MRI scans | 12 (11.11) | 0.168 | 0.015; 0.321 | **0.032*** | - | - | | - | 0.168 | 0.015;0.321 | **0.032*** |
| Any CT scans | 55 (50.93) | -0.057 | -0.206; 0.092 | 0.452 | - | - | | - | -0.057 | -0.206; 0.092 | 0.452 |
| Any emergency room visits* | 0 (0.00) | - | - | - | - | - | | - | - | - | - |
| Any radiation therapies | 1 (0.93) | -0.025 | -0.172; 0.122 | 0.738 | - | - | | - | -0.025 | -0.172; 0.122 | 0.738 |
| LOS** | - | 0.084 | -0.060; 0.228 | 0.254 | - | - | | - | 0.084 | -0.060; 0.228 | 0.254 |

Abbreviations: SD, standard deviation; CCI, Charlson Comorbidity Index; CI, confidence interval; CT, computed tomography; HCRU, health care resource utilization; ICU, intensive care unit; LOS, length of hospital stay; MRI, magnetic resonance imaging; PET, positron emission tomography; SD, standard deviation; SCT, stem-cell transplantation; USD, US dollars

*No variance in the variable, therefore, no effects observed

**Log link has been applied for total heathcare costs drivers in sem.

†Grouping subsequent therapy in full model (For example, if there is any one of DEVIC, CHASE, CAR T it will be included in the model with Therapy=1.)
