# Supplementary material for: Cost drivers associated with autologous stem-cell transplant (ASCT) in patients with relapsed/refractory diffuse large B-cell lymphoma in a Japanese real-world setting: A structural equation model (SEM) analysis 2012–2022
Source: PLoS One. 2025 Feb 6;20(2):e0317439. doi: 10.1371/journal.pone.0317439 (PMC11801729; doi:10.1371/journal.pone.0317439)
Supplement: S4 Table — (DOCX) [file pone.0317439.s004.docx]

**S4 Table:** **Direct, indirect, and total effects on healthcare cost obtained from Model 2**

| **Total health care cost drivers** | **N=108** | **Direct effects (USD)** | | | **Indirect effects (USD)** | | | **Total effects (USD)** | | |
| --- | --- | --- | --- | --- | --- | --- | --- | --- | --- | --- |
|  | **N (%)** | **β** | **95% CI** | **p** | **β** | **95% CI** | **p** | **β** | **95% CI** | **p** |
| **Patient characteristics** | | | | | | | | | | |
| Gender (reference: male) | | | | | | | | | | |
| Female | 45 (41.67) | -0.070 | -0.219; 0.079 | 0.357 | 0.108 | -0.019; 0.235 | 0.097 | 0.038 | -0.145; 0.220 | 0.687 |
| Age (reference: 18–65 years) | | | | | | | | | | |
| ≥66 years | 3 (2.78) | -0.005 | -0.150; 0.141 | 0.948 | -0.056 | -0.178; 0.067 | 0.375 | -0.060 | -0.243; 0.123 | 0.518 |
| Index year (reference: 2012–2019) | | | | | | | | | | |
| 2020–2022 | 34 (31.48) | 0.286 | 0.138; 0.434 | **<0.001***** | -0.104 | -0.238; 0.030 | 0.130 | 0.182 | 0.002; 0.362 | **0.048*** |
| **Comorbidities** | | | | | | | | | | |
| CCI score (reference: 0–2) | | | | | | | | | | |
| 3 | 17 (15.74) | 0.019 | -0.182; 0.220 | 0.855 | 0.067 | -0.103; 0.238 | 0.439 | 0.086 | -0.162; 0.334 | 0.496 |
| 4 | 28 (25.93) | 0.153 | -0.063; 0.369 | 0.166 | 0.040 | -0.144; 0.224 | 0.671 | 0.192 | -0.077; 0.462 | 0.162 |
| 5+ | 49 (45.37) | 0.191 | -0.055; 0.436 | 0.128 | 0.152 | -0.056; 0.361 | 0.153 | 0.343 | 0.055; 0.631 | **0.019*** |
| **Prior/concurrent non-lymphoma neoplasms (reference: No)** | | | | | | | | | | |
| Yes | 66 (61.11) | -0.072 | -0.227; 0.084 | 0.366 | 0.014 | -0.119; 0.148 | 0.833 | -0.057 | -0.249; 0.134 | 0.557 |
| **Complications** | | | | | | | | | | |
| Heart disease (reference: No) | 4 (3.70) | 0.143 | -0.010; 0.295 | 0.067 | - | - | - | 0.143 | -0.010; 0.295 | 0.067 |
| Kidney disease (reference: No) | 2 (1.85) | 0.029 | -0.113; 0.170 | 0.690 | - | - | - | 0.029 | -0.113; 0.170 | 0.690 |
| Liver disease (reference: No) | 8 (7.41) | -0.043 | -0.186; 0.100 | 0.556 | - | - | - | -0.043 | -0.186; 0.100 | 0.556 |
| **Chemotherapy regimen post-SCT** | | | | | | | | | | |
| Chemotherapy regimen post-SCT † | | | | | | | | | | |
| CAR T cell therapy (reference: No) | 2 (1.85) | 0.219 | 0.057; 0.380 | **0.008**** | 0.113 | 0.006; 0.221 | **0.039*** | 0.332 | 0.176; 0.488 | **<0.001***** |
| Any other chemotherapy (reference: No) | 13 (12.04) | 0.272 | 0.108; 0.436 | **0.001**** | 0.078 | -0.034; 0.190 | 0.171 | 0.350 | 0.194; 0.507 | **<0.001***** |
| **HCRU** | | | | | | | | | | |
| Number of hospitalizations | - | 0.370 | 0.218; 0.522 | **<0.001***** | - | - | - | 0.370 | 0.218; 0.522 | **<0.001***** |
| Any ICU admission (reference: No)* | 0 (0.00) | - | - | - | - | - | - | - | - | - |
| Any PET scans | 3 (2.78) | -0.003 | -0.146; 0.140 | 0.967 | - | - | - | -0.003 | -0.146; 0.140 | 0.967 |
| Any MRI scans | 12 (11.11) | 0.155 | 0.003; 0.307 | **0.046*** | - | - | - | 0.155 | 0.003; 0.307 | **0.046*** |
| Any CT scans | 55 (50.93) | -0.055 | -0.203; 0.093 | 0.470 | - | - | - | -0.055 | -0.203; 0.093 | 0.470 |
| Any emergency room visits* | 0 (0.00) | - | - | - | - | - | - | - | - | - |
| Any radiation therapies | 1 (0.93) | -0.023 | -0.169; 0.124 | 0.764 | - | - | - | -0.023 | -0.169; 0.124 | 0.764 |
| LOS** | - | 0.114 | -0.035; 0.262 | 0.133 | - | - | - | 0.114 | -0.035; 0.262 | 0.133 |

Abbreviations: SD, standard deviation; CCI, Charlson Comorbidity Index; CI, confidence interval; CT, computed tomography; HCRU, health care resource utilization; ICU, intensive care unit; LOS, length of hospital stay; MRI, magnetic resonance imaging; PET, positron emission tomography; SD, standard deviation; SCT, stem-cell transplantation; USD, US dollars

*No variance in the variable, therefore, no effects observed

**Log link has been applied for total healthcare costs drivers in sem.

†Reference grouping is different from previous study.
