# Supplementary material for: Cost drivers associated with autologous stem-cell transplant (ASCT) in patients with relapsed/refractory diffuse large B-cell lymphoma in a Japanese real-world setting: A structural equation model (SEM) analysis 2012–2022
Source: PLoS One. 2025 Feb 6;20(2):e0317439. doi: 10.1371/journal.pone.0317439 (PMC11801729; doi:10.1371/journal.pone.0317439)
Supplement: S5 Table — (DOCX) [file pone.0317439.s005.docx]

**S5 Table: Geographical distribution of hospital facilities conducting ASCT**

| **Hospital facility regions** | **n (%)** |
| --- | --- |
| Hokkaido | 2 (1.85) |
| Tohoku | 10 (9.26) |
| Kanto | 42 (38.89) |
| Chubu | 21 (19.44) |
| Kinki | 12 (11.11) |
| Chugoku | 6 (5.56) |
| Shikoku | 3 (2.78) |
| Kyushu | 12 (11.11) |
