# Supplementary material for: Cost drivers associated with autologous stem-cell transplant (ASCT) in patients with relapsed/refractory diffuse large B-cell lymphoma in a Japanese real-world setting: A structural equation model (SEM) analysis 2012–2022
Source: PLoS One. 2025 Feb 6;20(2):e0317439. doi: 10.1371/journal.pone.0317439 (PMC11801729; doi:10.1371/journal.pone.0317439)
Supplement: S6 Table — (DOCX) [file pone.0317439.s006.docx]

**S6 Table: Total health care costs as per patient per year in JPY and USD**

|  | **JPY** | | | | **USD** | | | |
| --- | --- | --- | --- | --- | --- | --- | --- | --- |
| **N = 108** | **Cost before adjustment** | | **Cost after adjustment** | | **Cost before adjustment** | | **Cost after adjustment** | |
| Mean, SD | ¥10,466,478.47 | ¥16,064,309.33 | ¥10,466,478.47 | ¥16,064,309.33 | $78,671.67 | $120,747.97 | $79,052.44 | $121,503.65 |
| Median | ¥5,693,100.07 | - | ¥5,693,100.07 | - | $42,792.39 | - | $42,722.82 | - |
| Q1, Q3 | ¥2,846,515.22 | ¥9,806,123.34 | ¥2,846,515.22 | ¥9,806,123.34 | $21,395.94 | $73,708.08 | $21,475.21 | $73,722.68 |
| Min, Max | ¥684,406.71 | ¥94,077,605.43 | ¥684,406.71 | ¥94,077,605.43 | $5,144.37 | $707,137.74 | $5,166.36 | $705,239.70 |

Abbreviation: SD: Standard deviation; Q1: First quartile; Q3: Third quartile
