# Supplementary material for: Cost drivers associated with autologous stem-cell transplant (ASCT) in patients with relapsed/refractory diffuse large B-cell lymphoma in a Japanese real-world setting: A structural equation model (SEM) analysis 2012–2022
Source: PLoS One. 2025 Feb 6;20(2):e0317439. doi: 10.1371/journal.pone.0317439 (PMC11801729; doi:10.1371/journal.pone.0317439)
Supplement: S7 Table — (DOCX) [file pone.0317439.s007.docx]

**S7 Medical fee price index for recent medical service fee revisions**

| **Period** | **Adjustment rate for the fiscal year 2022 cost[31]** | |
| --- | --- | --- |
|  | **Medical treatment** | **Drug** |
| Apr 2012 - Mar 2014 | 1.033 | 0.939 |
| Apr 2014 - Mar 2016 | 1.025 | 0.944 |
| Apr 2016 - Mar 2018 | 1.019 | 0.956 |
| Apr 2018 - Sep 2019 | 1.013 | 0.972 |
| Oct 2019 - Mar 2020 | 1.008 | 0.977 |
| Apr 2020 - Mar 2022 | 1.003 | 0.987 |
| Apr 2022 - Current | 1.000 | 1.000 |
